# An easy, flexible solution to attach devices to hedgehogs (*Erinaceus europaeus*) enables long‐term high‐resolution studies

**DOI:** 10.1002/ece3.4794

**Published:** 2018-12-21

**Authors:** Leon M. F. Barthel, Heribert Hofer, Anne Berger

**Affiliations:** ^1^ Department of Evolutionary Ecology Leibniz Institute for Zoo and Wildlife Research (IZW) Berlin Germany; ^2^ Berlin‐Brandenburg Institute of Advanced Biodiversity Research Leibniz Institute for Zoo and Wildlife Research (IZW) Berlin Germany

**Keywords:** accelerometer, attachment, biotelemetry, device, *Erinaceus*, GPS, long‐term measurement

## Abstract

Bio‐logging is an essential tool for the investigation of behavior, ecology, and physiology of wildlife. This burgeoning field enables the improvement of population monitoring and conservation efforts, particularly for small, elusive animals where data collection is difficult. Device attachment usually requires species‐specific solutions to ensure that data loggers exert minimal influence on the animal’s behavior and physiology, and ensure high reliability of data capture. External features or peculiar body shapes often make securing devices difficult for long‐term monitoring, as in the case with small spiny mammals. Here, we present a method that enables high‐resolution, long‐term investigations of European hedgehogs (*Erinaceus europaeus*) via GPS and acceleration loggers. We collected data from 17 wild hedgehogs with devices attached between 9 and 42 days. Our results showed that hedgehogs behaved naturally; as individuals curled, moved through dense vegetation, slipped under fences and built regular day nests without any indication of impediment. Our novel method makes it possible to not only attach high‐precision devices for substantially longer than previous efforts, but enables detachment and reattachment of devices to the same individual. This makes it possible to quickly respond to unforeseen events and exchange devices, and overcomes the issue of short battery life common to many lightweight loggers.

## 1 INTRODUCTION

A thorough knowledge of the ecology, behavior, and physiology of species under free‐ranging conditions is essential to understand their environmental needs, life‐history strategies and thus is the crucial basis for their protection and conservation (Kays, Crofoot, Jetz, & Wikelski, 2015; LaPoint, Balkenhol, Hale, Sadler, & van der Ree, 2015; Wilson & McMahon, 2006). Research on movement patterns, habitat use, interspecies and intraspecies interactions, foraging and reproductive behavior is essential for effective conservation management (Fraser et al., 2018; Graham, Douglas‐Hamilton, Adams, & Lee, 2009). Such research, benefits from high resolution, long‐term data collection and can help develop effective nature reserves (Afonso, Fontes, Holland, & Santos, 2008), solve human‐wildlife conflicts (Voigt et al., 2014) and improve captive breeding to ensure successful re‐introduction of endangered species (Kaczensky et al., 2011). This field of “big‐data animal tracking” is advancing with the development of lightweight bio‐logging devices capable of combining accelerometer, VHF and/or GPS (Kays et al., 2015).

The results of studies on behavior and physiology of wildlife under controlled conditions can often not be reproduced under natural conditions, making studies on free‐ranging animals in their native habitat crucial (Gattermann et al., 2008). Unfortunately, field studies can be difficult as observer presence is known to influence animal behavior; however, subtle the observation conditions might be (Cagnacci, Boitani, Powell, & Boyce, 2010; Crofoot, Lambert, Kays, & Wikelski, 2010; Kays et al., 2015; Scheibe & Gromann, 2006; Schneirla, 1950; Shamoun‐Baranes et al., 2012). Innovative biotelemetry/bio‐logging technologies are being applied to an ever‐increasing range of taxa (from insects to mammals), spatial scales (from habitat patch to continental scale), and habitats (from coral reefs to rainforests) enabling us to gain a deeper insight into the natural behavior and physiology of species without the need for observer presence (Cagnacci et al., 2010; Kays et al., 2015; Wilson & McMahon, 2006). Yet, these devices may also change the behavior of the animals to which they are attached, or may influence their chance of survival, thereby also biasing results (e.g., Hofer & East, 1998). In order to reduce such biases, the data logger, attachment and handling procedure should all minimize disturbance of the study animals (Barron, Brawn, & Weatherhead, 2010; Collins, Petersen, Carr, & Pielstick, 2014; Hofer & East, 1998; Pearl, 2000; Vandenabeele et al., 2015). Therefore devices should be designed in a species‐specific manner. Such a design needs to take into account the mass, size, shape, and material of the device and the method of its attachment and potential for detachment/reattachment (Bridge et al., 2011; Culik, Bannasch, & Wilson, 1994; Kays et al., 2015; Vandenabeele, Wilson, & Wikelski, 2013).

Concerning the mass of the device, it is recommended that a complete radio transmitter should not exceed 2%–5% of body mass (Hofer & East, 1998; Kenward, 2001; Sikes & Gannon, Animal Care and Use Committee, 2011). Despite the wide acceptance of the “percentage rule,” a meta‐analysis of bird behavioral studies found little evidence that the impact of carrying the device was proportional to its weight (Barron et al., 2010). In contrast, in a study of equids, Brooks, Bonyongo, and Harris (2008) showed that, even within the accepted norms, small differences in collar mass can significantly affect specific behaviors. Regardless, both studies found that attachment position and collar fit impacted behaviors significantly (Barron et al., 2010; Brooks et al., 2008). A key issue is that battery mass and size are the driving factors of device total mass. Together they determine battery life and thus the duration of data collection. The trade‐off between light mass and long duration is particularly challenging when the study species are small, such as many lizards, birds or small mammals (Dervo et al., 2010; Doody, Roe, Mayes, & Ishiyama, 2009; Flesch, Duncan, Pascoe, & Mulley, 2009; Rautio, 2015; Warner, Thomas, & Shine, 2006; Warwick, Morris, & Walker, 2006).

While battery life and device size pose substantial hurdles for study design, data retrieval is by far the most important aspect of any study. The current methods for device attachment dictate that devices fall off upon glue deterioration and/or after the growth of fur or feathers or the shedding of spines. Yet this approach may not always be viable and animal recapture and manual device removal is also commonly necessary. As such, the swift and easy removal and reattachment of devices enables data download, battery exchange and the potential for prolongation of data collection. While solutions to battery life via solar powered devices have enabled long‐term data collection in diurnal species, nocturnal animals are still considered elusive and bio‐logging study design must be approached differently.

European hedgehogs (*Erinaceus europaeus*) are a small, nocturnal mammal (ranging seasonally from 600 to 1,500 g) with a highly flexible body covered in spines. These spines are made of keratin and are repeatedly shed during a hedgehog's lifetime. Individuals hide and forage in dense vegetation and have a number of interesting behaviors such as self‐anointing, curling up, hibernation, and regular nest building (Hof, 2009; Reeve, 1994; Reeve, Bowen, & Gurnell, 2015). Because of these characteristics and their unusual body shape, standard collars cannot be used and other methods of attachment are often unsuitable. For a more comprehensive understanding of hedgehog behavior longer‐term data sets are extremely important and contingent on appropriate device selection and attachment.

Here, we present a modified method of device attachment to hedgehogs that does not hinder the animal's movements and can be easily removed and replaced, thereby solving the trade‐off between small device size and the collection of long‐term high‐resolution data.

## 2 METHODS

### 2.1 Study area and animals

Fieldwork was conducted from August to September 2016 in a study area of 16 ha within an urban park of 88.2 ha, in southeast Berlin, Germany (52.48846°N, 13.46974°E) as part of an ongoing project. The park is open to the general public and comprises short grass, variable shrub density, gravel foot paths, a playground, and a monument site. The park is surrounded by urban pedestrian areas, tarmacked streets and parking areas to the east and south, and is bounded by the river Spree to the north and by a railway embankment to the west. The park was open to the general public throughout day and night.

When traversing this urban park, hedgehogs may have to cross streets, slip through fences or climb up a railway embankment. In preparation of this study, three night surveys to find hedgehogs were carried out at least one hour after sunset to find the animals by spotlighting (P14.2, LED Lenser, Solingen, Germany). Every hedgehog was marked with five labelled shrink tubes on the spines (Mori, Menchetti, Bertolino, Mazza, & Ancillotto, 2015). The tubes were labelled with a number starting with 1 to make it possible to identify them during recapture (N. J. Reeve, pers. comm. 2016).

### 2.2 Backpack attachment

**Figure 1:**
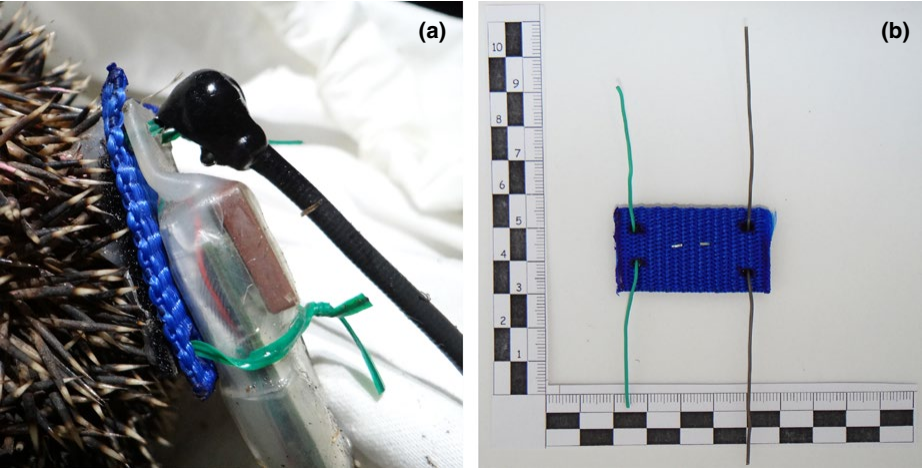
(a) A complete backpack glued to the hedgehog's spines including the back plate consisting of firmly glued fabric (blue) and loop strap (black below the blue fabric), the wires (green), the GPS device (transparent shrink tube), and the VHF transmitter (black). (b) The back plate system from above; scale numbers indicate cm. Photograph: Leon M. F. Barthel

The complete backpack comprised three components: the back plate, the data logger (GPS and accelerometer), and a very high frequency (VHF) transmitter (Figure 1a).

The back plate consisted of 2.5 cm wide and 1.6 mm thick fabric material, a synthetic woven material made from polyethylene often used for belts, cut into 4.5 cm long strips. Four holes were burned into this fabric using a soldering iron to facilitate entry of two wires of different length (7.5 cm and 10 cm) (Figure 1b). These wires were later used to fasten the devices (datalogger and transmitter) to the back plate (Figure 1b) and could be used to easily attach or remove different devices to and from the plate. Some of the VHF transmitters had small tubes attached to them; therefore, it was easy to just insert the wires. Others had to be glued to a different spot directly between the spines. For fixation wires were twisted, trimmed to length and bent in such a way that they were aligned with the devices to prevent entangling or poking the hedgehogs. We tested different wires (steal, isolated copper, florist's wire) of which the isolated copper wire with a 1 mm diameter turned out to be best as it lasted longer. After inserting the wires through the fabric from below, a piece of soft Velcro (loop strap, 2.5 × 4.5 cm in size) was glued to the lower surface of the fabric, thereby fixing the wires in place and maximizing the surface available for the attachment of the complete backpack to the animal. The connection of fabric and Velcro could be strengthened if necessary using a paperclip or hot glue.

To reduce costs, the data loggers were manually put together using components supplied by eobs‐GmbH (www.e-obs.de, Gruenwald, Germany) or CellGuide Ltd. (www.cell-guide.com, Netanya, Israel). The circuit boards for GPS and acceleration measurements were obtained from e‐obs GmbH and were combined with and soldered to lithium‐poly‐accumulators of two different capacities (260 mAh or 300 mAh at 3.7 V) and cased in heat shrink tubes of 46 mm width. Sealing with hot glue at the ends of the heat shrink tubes ensured waterproof packaging. Covering the terminal poles used for recharging with hot glue prevented the establishment of creeping currents in the field. These custom‐built loggers had a total mass of between 19.09 g and 20.36 g (e‐obs GmbH) or between 11.97 g and 12.83 g (CellGuide Ltd.).

In this study, we used several different models of VHF transmitters of varying weight. Transmitters were supplied by the companies “Andreas Wagner” (www.wagener-telemetrie.de, weight ~4 g), and “TELEMETRIE‐SERVICE DESSAU” (www.telemetrie-service.de, Dessau, Germany, weight 4 or 11 g), and we also custom built our own devices (weight ~11 g). All transmiters sent a simple short signal (150 MHz) for up to several months depending on the battery size. With the Wagener and 11 g Telemetrie‐Service Dessau models, it was possible to insert the wire in a tubing at the base of the VHF transmitter (Figure 1a). Our custom made devices had wires attached that were twisted with the wires on the back plate and the 4 g transmitters of Telemetrie‐Service Dessau were glued directly between the spines.

### 2.3 Fitting and removing of the backpack

After capture during night surveys, and before attaching the backpack, the hedgehogs were sexed and weighed (while held inside a cloth bag) using a hanging scale (HDB 5K5N, Kern & Sohn GmbH, Balingen, Germany, weighing accuracy 5 g). The base plate was only attached to healthy hedgehogs with a minimum mass of 600 g. Approximately 3 mm was cut from the tips of the spines using scissors to provide a larger contact area for attachment. This procedure is harmless because spines are made of keratin throughout and do not contain nerves or blood vessels. In contrast to previous studies of hedgehogs (Abu Baker, Reeve, Conkey, Macdonald, & Yamaguchi, 2017; Abu Baker et al., 2016; Braaker et al., 2014; Pettett, Johnson, et al., 2017; Pettett, Moorhouse, Johnson, & Macdonald, 2017; Reeve, 1997; Warwick et al., 2006), we used hot glue to attach the back plate because a mobile hot glue gun (neo1, Steinel Vertrieb GmbH, Herzebrock‐Clarholz, Germany) can be used very quickly and precisely and is cost effective. Hot glue sets within seconds and is therefore much faster and deliquesces much less than other commonly used and in this project previously tested glues and epoxies. The hot glue was applied across the complete underside of the back plate, with a thickness of about 3 to 4 mm and then pressed into the spines, ensuring that the glue surrounded all spine tips. Sometimes it was necessary to add glue from the side as well. During this procedure, we ensured that the hot glue did not come into contact with the hedgehogs's skin.

The back plate was placed on the hedgehog body at the same location as described by Recio, Mathieu, and Seddon (2011), around two‐thirds along the center of the main body on its back distal to the head. Data loggers were attached to the back plate by inserting the wires in holes on the device. The longer wires were screwed tightly around the device to fix it to the back plate and prevent loosening and wobbling. On one of the wires, a VHF transmitter (with holes) could be attached to locate the hedgehogs in the field or directly clued between the spines if the model had no tubing to insert the wire. After twisting, we trimmed the wires and aligned the wires with the logger. Thus, the combination of short wires aligned to the logger, a spine length away from the body ensured that the hedgehogs were not poked. After attaching the backpack, animals were re‐weighed. Initial handling took a maximum of 10 min, including sexing and weighing the animal, cutting the spines and gluing the back plate onto the back of the hedgehog.

After the devices were attached, we located individual hedgehogs by their individual logger frequency with the help of a receiver (TRX‐1000S, Wildlife Materials Inc., Murphysboro, IL, USA, or Wide Range Receiver AR 8200, AOR Ltd., Tokyo, Japan). Hedgehogs were tracked, recaptured and checked every day to detect whether they behaved normally or had problems to build their nests or overcome obstacles. Additionally, once a week all hedgehogs were weighed and inspected for any problems. At the very end of the experiment, the back plate was cut off the spines below the hardened hot glue, to leave as much a length of spines as possible to ensure that the skin was not bare. In order to continue monitoring of individuals until the beginning of hibernation, we then glued another small VHF unit (~ 4 g) onto the spines using again hot glue.

### 2.4 Statistical analyses

Eighteen hedgehogs (8 females, 10 males) were initially fitted with devices for a total time of deployment of between 9 days and 42 days (Table 1). One hedgehog (ID15) was not included in the data analysis because it was found dead just two days after transmitter attachment after it was run over by a train. Thus, results are presented for 17 hedgehogs. To compare differences in hedgehog body masses recorded prior to the attachment and after the removal of the back plate, a Wilcoxon signed‐ranks test with continuity correction was conducted. The test was performed in R version 3.4.2 using the core package (R Core Team, 2016). Results are reported as means ± standard deviation (*SD*).

**Table 1:** Characteristics of all study animals: ID, sex, body mass at start and end of the experiment, body mass change number of deployments, duration of complete deployment, and, for illustrative purposes, the relative mass for a 30 g back plate device combination

Animal ID	Sex	Body mass [g]	Body mass [g]	Body mass, change [g]	Number of deployments	Duration of deployment [days]	Relative mass of the heaviest backpack [%]	
		Start	End				Start	End
2	f	1,090	935	−155	4	41	2.7	3.2
7	f	1,085	1,005	−80	3	41	2.7	2.9
8	f	795	835	40	4	41	3.7	3.5
9	f	830	1,010	180	4	41	3.6	2.9
13	f	725	885	160	4	41	4.1	3.3
16	f	890	1,030	140	1	40	3.3	2.9
17	f	1,480	1,015	−465	4	41	2.0	2.9
20	f	1,100	990	−110	1	40	2.7	3.0
1	m	1,060	1,095	35	4	41	2.8	2.7
5	m	840	850	10	1	20	3.5	3.5
10	m	1,180	865	−315	0	9	2.5	3.4
11	m	900	1,005	105	2	36	3.3	2.9
14	m	770	980	210	1	41	3.9	3.0
15	m	990	dead	na	0	2	3.0	na
18	m	935	1,145	210	1	41	3.2	2.6
19	m	890	990	100	4	41	3.3	3.0
21	m	1,015	1,340	325	4	41	2.9	2.2
22	m	940	1,090	150	0	28	3.1	2.7

## 3 RESULTS

The complete backpack system once attached to the animals weighed between 25 g and 31 g; this depended on the device and the amount of glue used. The body mass of hedgehogs varied between 725 g and 1,480 g (mean 972.1 ± 184.7 g, *n* = 17, Table 1), resulting in a relative mass of the complete backpack below 4.2% of body mass. Hedgehogs with attached devices slipped under fences and crossed dense vegetation (e.g., *Hedera helix*, *Humulus lupulus*) and regularly built new nests without showing any negative effects from the backpack system. During early trials, single spines were pulled out of the skin by the load of the backpack. This problem disappeared after applying more glue to surrounding spines near the plate.

During the study, the backpack or parts of it detached themselves on three occasions. From one hedgehog (ID 5), the backpack system had to be removed after 20 days due to dirt and the presence of fly larva under the plate. Later on, this hedgehog was recaptured twice identified by the yellow ID tubes and we observed that it had recovered completely and gained weight within two weeks. Because the field experiment had been completed, no reattachment was considered. One device was found with markings similar to that from canine teeth, indicating that maybe a predator had caught the hedgehog. Yet this individual was able to escape and was later re‐caught by us and the device was reattached. In the third case, a device was found in an open meadow, including the spines to which it was glued; there was no visible reason for this detachment. The hedgehog was found later that day alive and well and the device was reattached.

**Figure 2:**
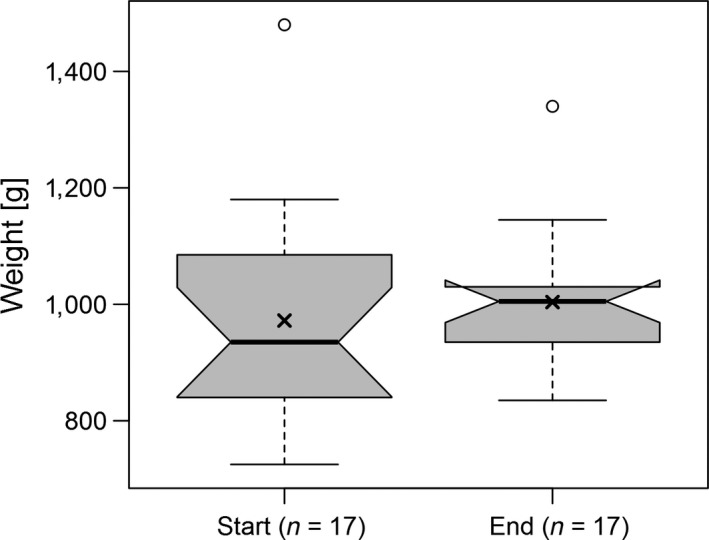
Boxplots of body mass of hedgehogs on the first day of deployment (Start) and on the last day of deployment (End). Central line: median, *x*: location of mean, whiskers: 1.5 times the interquartile range, circle: values more extreme than 1.5 times interquartile range around the median

Attachment and removal of GPS devices worked well as the process was swift and easy. Four backpacks had to be repaired while attached to the hedgehog, which took about 5 to 10 min of wire replacement and application of additional glue. After removing the back plate, the body mass of hedgehogs ranged from 835 g to 1,340 g (mean 1,004 ± 122.7 g *n* = 17, Figure 2). Another hedgehog (ID 10) was found freshly dead, so we took the mass and used it in the analyses. There was no difference in body mass between the start and the end of device deployment of the hedgehogs (Wilcoxon signed‐ranks test with continuity correction, *V* = 100, *p* = 0.28). Twelve out of 17 animals gained weight during the device deployment period. One male (ID 10) and four females (ID 2, 7, 17, 20) lost weight. This male (ID 10) was found dead on the ninth day of the experiment; the necropsy confirmed that this individual was infected by lungworms which might have already had an impact on its health and behavior before the device had been attached. The area below the backpack of this animal showed no signs of infection.

During the study, four females (ID 2, 7, 17, 20) gave birth to hoglets, in three cases confirmed by sightings near the nest (ID 2, 7, 20) and/or by the increase in the size of teats of females (ID 2, 7, 17, 20). One female (ID 17) showed unusual behavior in terms of restlessly moving during the whole night and during some days and died a few days before the study ended.

## 4 DISCUSSION

European hedgehogs are an excellent example of an elusive species where data on behavior, movement, and ecology is essential for appropriate conservation management. While the UK, Sweden, and Denmark report alarming decreases in hedgehog populations, other countries cannot provide population sizes or trends because the effort required to adequately monitor hedgehogs cannot currently be undertaken (Hof, 2009; Hof & Bright, 2009; Huijser & Bergers, 2000; Johnson et al., 2015; Krange, 2015). To date, data collection on free‐ranging individuals has been limited to VHF tracking or short term GPS studies, primarily due to issues of device design and attachment. Our design provides a novel way of tackling these problems using cheap and effective materials to enable long‐term monitoring.

Here, we used fabric material for the ground plate which was cheap, is widely available and sufficiently robust for long‐term outdoor use. It is elastic, durable, breathable, and easy to work with. If necessary, the color could be suitably chosen to avoid making the animal conspicuous and more interesting for potential predators (the oddity effect, for example; Beauchamp, 2014). Isolated copper wire of 1 mm diameter proved to be most suitable as it was the most flexible, light weight wire that was also durable; facilitating repeated attachments.

Previous studies have commonly used fast curing epoxy for the attachment of devices to hedgehogs (Abu Baker et al., 2017, 2016; Bontadina, 1991; Braaker, 2012; Braaker et al., 2014; Braaker, Obrist, Bontadina, & Moretti, 2012; Esser, 1984; Pettett, Johnson, et al., 2017; Pettett, Moorhouse, et al., 2017; Reeve, 1997; Warwick et al., 2006). However, the hot glue we used was more suitable to fix the back pack on the hedgehog's spines as it was easy, cheap, and fast curing. We have had no problem in applying the glue using a small mobile glue gun, and in no case did the glue reach the skin and thus did not risk injury of the animals. Previous extensive tests of different glues and resins (Esser, 1984; Zingg, 1994) already demonstrated that epoxies suffer from long curing times, emit aerosols, generate high temperatures, and require additional material to protect the animal. The only disadvantage of hot glue may be that it may not work properly if used in very wet weather conditions. We do not have enough experience with the dental composite used by Reading, Kenny, Murdoch, and Batdorj (2016) to compare its characteristics and handling with hot glue. At 225 US$ for the initial application to 10 hedgehogs and 140 US$ for refills, dental composite is much more costly than hot glue (~ 55 US$ for 17 hedgehogs for the initial application, 0 US$ for exchanging devices on the back plate).

Our study resulted in a substantially longer duration of logger deployment than other GPS studies on hedgehogs at 42 days compared with the previous 8 days (Abu Baker et al., 2017; Braaker et al., 2014; Glasby & Yarnell, 2013; Recio et al., 2011). From our personal knowledge of many other attachment systems, the system we describe here is smaller and also enables a quick and easy exchange of data loggers, from small sensors for light, temperature, acceleration or noise to relatively heavy GPS‐loggers. The major improvement is the higher flexibility when attaching and removing devices. Other studies of hedgehogs did not reattach GPS devices to animals (Abu Baker et al., 2017) or they focused on the replacement of batteries (Boitani & Reggiani, 1984). For example, Braaker et al., (2014) reported that they reattached their devices but did not provide any details on the method. This may therefore be the first time that a fast and easy replacement of GPS data loggers on a fixed back plate has become possible, thereby enabling long‐term and high‐resolution studies of hedgehogs. In our study, we were able to detach and reattach rechargeable devices with short battery lifetimes in order to extend data collection. Moreover, our system provided the option of flexible solutions for potentially sensitive periods such as the mating season or lactation period, during which the behavior, reproductive success or health of the animal might be negatively influenced by cumbersome devices. For these periods, such devices could be replaced by small and light VHF transmitters which provide the opportunity to continue monitoring the animal. Furthermore, our system permits a fast response to unforseen situations.

Why do hedgehogs lose attachments? We suspect that the constant drag on the spines could lose either the attachment or the spine. The skin may release a single spine at any time. This may increase bending forces applied by body movements, accelerating the subsequent loosening of spines or the attachment. Such bending moments could be particularly strong that when animals curl up as then bending forces would be at a maximum.

Our mode of attachment permits short handling times and removes the need for anesthesia. With a little bit of experience, the complete time for the initial deployment is less than 10 min. The checking and exchange of loggers on a deployed back plate took less than 1 min, including the measurement of body mass. This is amongst the fastest handling times which we are aware of and minimizing this time is desirable to reduce stress on the animal.

For hedgehogs, as small hibernating insectivores, body mass is an essential feature for assessing individual survival and fitness. Yet fluctuations in body mass can be swift and may even simply result from variable foraging success. For example, hedgehogs can increase their mass following feeding by 20 g in as little as two hours (Rautio, Valtonen, & Kunnasranta, 2013). While mass gains of up to 157 g have been reported within one night through multiple feeding events (Morris, 1985). We considered the change in body mass during our study period as a possible biomarker to assess to what extend the animals reacted to the backpack—unusually large loss of body mass could be an indicator of stress or disturbance of natural behavior (Boitani & Reggiani, 1984; Kristiansson, 1984; Recio et al., 2011). Our modified back plate had a mass of ~2 g. Thus, our method permitted the attachment of devices with a mass of a maximum of ~28 g in order to stay below the recommended limit of 5% body mass (Sikes & Gannon, 2011), since we stipulated that hedgehogs could only be tagged if their body mass exceeded 600 g.

In our study, we observed weight losses by 5 of 17 individuals (one male and four females), with an average mass loss of 225 g (range 80–465 g). For the male hedgehog (ID 10), an infection with lungworm may have exacerbated the challenges posed by the mating season and therefore instigated substantial weight loss resulting in his eventual death. Alternatively, long‐term stress may have exacerbated the infection with lungworm, compromising immunocompetence (cf. a similar argument for lactating females and gastrointestinal hookworm burdens in East et al., 2015). Weight loss for all four females was most likely associated with giving birth and maternal care of hoglets as three of the four females were found with hoglets litters during the study. With animal ID 17, there were no confirmed hoglet sightings although she showed teats increased in size. The mass of hoglets at birth varies between 8 g and 25 g (Burton, 1969; Herter, 1965; Morris, 1977; Versluys, 1975) and, with an average of four hoglets per litter, there is a prospective mean weight loss of 24 g to 100 g per female. In addition, the energetically costly period of lactation results in rapid mass fluctuations for female hedgehogs (Kristiansson, 1984; Rautio et al., 2013). Considering that these individuals were able to give birth and continued to care for their litters until the hoglets successfully left the nest, suggests that the life‐history of these individuals was not substantially affected by our devices.

The placement of the system on the animal's back enabled hedgehogs to move unhindered, as they were found to undergo normal behavior of crawling under fences and through dense vegetation. While dirt accumulated under the backpack, with regular checks of study animals any negative consequences could be prevented. This will also help to identify any possible physical deterioration from stressful responses (which we did not observe in our study) to the repeated deployment and attachment of recording devices. Since our study took place after the mating season, we do not know whether the backpack impedes mating behavior and copulation, so this still needs to be clarified. Overall, our results demonstrate that the backpack had little influence on study animal behavior. However, we still suggest regular recapture of individuals to mitigate any potential negative consequences to welfare. In conclusion, we present an improved method for the attachment and reattachment of bio‐logging technology to small mammals with a unique body structure.

## CONFLICT OF INTEREST

None declared.

## AUTHOR CONTRIBUTIONS

LB, AB, and HH conceived and designed the experiments. LB performed the experimentsand analyzed the data. AB and HH contributed reagents/materials/analysis tools. LB wrote the first draft of the manuscript. AB and HH read and improved the manuscript. All authors contributed critically to the drafts and gave final approval for publication.

## DATA ACCESSIBILITY

All data used in this manuscript are in the original text and tables.
